# Mice lacking fat storage-inducing transmembrane protein 2 show improved profiles upon pressure overload-induced heart failure

**DOI:** 10.1016/j.heliyon.2019.e01292

**Published:** 2019-03-13

**Authors:** Natsumi Nishihama, Takahiro Nagayama, Shinji Makino, Ryuta Koishi

**Affiliations:** a Venture Science Laboratories, R&D Division, Daiichi Sankyo Co., Ltd., 1-2-58, Hiromachi, Shinagawa-ku, Tokyo 140-8710, Japan; b Rare Disease Laboratories, R&D Division, Daiichi Sankyo Co., Ltd., 1-2-58, Hiromachi, Shinagawa-ku, Tokyo 140-8710, Japan; c Department of Cardiology and Health Center, Keio University School of Medicine, Shinano-machi 35, Shinjuku-ku, Tokyo 160-8582, Japan

**Keywords:** Cardiology, Physiology

## Abstract

Fat storage-inducing transmembrane proteins 1 and 2 (FITM1 and FITM2, respectively) are transmembrane endoplasmic/sarcoplasmic reticulum proteins involved in lipid droplet formation. The physiological functions of FITM1 have only been reported in skeletal muscle, while those of FITM2 were analyzed using genetically engineered mice. However, their roles in the heart have not been characterized. To examine their cardiac functions, we analyzed *Fitm1-* or *Fitm2*-knockout mice. Neither constitutive *Fitm1* (−/−) aged nor heart failure model mice showed significant differences in heart size or function. *Fitm2* (−/−) mice exhibited embryonic death, and aged *Fitm2* (+/−) mice had shortened left ventricular end-diastolic dimension, and shortened left ventricular end-systolic dimension. However, body weight and ejection fraction of *Fitm2* (+/−) mice were similar to those of wild-type littermates. In the chronic heart failure models, *Fitm2* (+/−) mice showed significant suppression of increased left ventricular end-diastolic dimension and reduced ejection fraction. These results suggest the involvement of *Fitm2* in chronic heart failure, whereas *Fitm1* have a minor effect in this context in mice.

## 1 Introduction

Fat storage-inducing transmembrane proteins 1 and 2 (FITM1 and FITM2, respectively) are members of a conserved gene family that are important for lipid droplet accumulation [1, 2, 3]. Both human *FITM1* and *FITM2* are highly expressed in oxidative tissues such as the heart and skeletal muscle; specifically, *FITM1* is highly expressed in skeletal muscle, whereas *FITM2* is primarily expressed in the heart. In mice, *Fitm1* and *Fitm2* exhibit mRNA expression patterns similar to those of humans, but Fitm2 protein is most highly expressed in adipose tissues [1].

On the basis of the expression pattern of FITM1, its functions were analyzed during different developmental stages of pig skeletal muscle and cultured cells. Porcine *Fitm1* gene expression changes during skeletal muscle development and is regulated by MyoD1 in C2C12 cells during differentiation [4]. In addition, overexpression of peroxisome proliferator-activated receptor gamma coactivator 1-alpha (PGC-1α) induces *FITM1* expression in cultured human skeletal muscle cells with the accumulation of lipid droplets [5].

Meanwhile, the physiological functions of FITM2 were analyzed using adipose-specific-knockout mice [6], postnatal whole-body-knockout mice [7], and skeletal muscle-specific-overexpressing mice (CKF2) [8]. These studies indicate that FITM2 has essential roles in normal fat storage and intestinal health. In addition, *Fitm2* overexpression leads to decreased fatty acid oxidation, increased utilization of branched-chain amino acids, enhanced glucose uptake, and profoundly decreased cellular ATP levels in the skeletal muscle of CKF2 mice. These findings also indicate that Fitm1 and Fitm2 have important roles in skeletal muscle. Considering that both skeletal muscle and the heart are oxidative tissues, and obtained findings on the patterns of expression of FITM1 and FITM2, previous results suggest that these two molecules may have roles in the heart.

Recently, we found that these proteins were identified in mice and/or human heart samples by proteomic analysis, affected the cardiovascular system and body development in zebrafish, and that their modulated expression or function can change lipid droplet formation, ER function, and cellular metabolism in cells [9]. These data suggested their potential to modulate heart failure pathogenesis. However, their physiological functions in heart have not been elucidated.

Therefore, we here report the profiles of FITM1 and FITM2 in mouse models of heart failure. The mRNA expression of *Fitm1* and *Fitm2* was decreased in heart failure model mice, and *Fitm2* hetero-knockout [*Fitm2* (+/−)] mice exhibited resistant profiles to pressure-induced chronic heart failure. However, neither *Fitm1* (−/−) nor *Fitm2* (+/−) status negatively affected cardiac development or function in normal conditions. Our findings collectively suggest that downregulation of *Fitm*, especially *Fitm2* expression or function may have a beneficial effect in patients with heart failure.

## 2 Materials and methods

### 2.1 Animals

Seven-week-old male C57Bl/6 mice were purchased from Japan SLC, Inc. *Fitm1-* or *Fitm2*-knockout mice were generated at the Institute of Immunology Co., Ltd. (formerly Phoenix Bio). Seven- or eight-week-old male *Fitm1* (−/−), *Fitm2* (+/−), and littermate mice were purchased from the Institute of Immunology Co., Ltd. The mice were kept in a room at 55% relative humidity (permissible range: 30%–70%) and 23 °C (permissible range: 20–26 °C) under a 12-h light/dark cycle. The animal experiments were carried out in accordance with the guidelines of the local Institutional Animal Care and Use Committee and were reviewed and approved by it.

### 2.2 Animal models and cardiac function assessment

At nine weeks of age, mice were intubated and anesthetized with isoflurane gas. For the chronic heart failure model induced by pressure overload, the transverse aorta was exposed by median incision, and pressure overload was produced by transverse aortic constriction (TAC) using a 27-gauge needle. For the chronic heart failure model induced by myocardial infarction (MI), the chest cavity was exposed by cutting the intercostal muscle, and the left coronary artery was subsequently ligated with a 7–0 silk suture. To generate sham-operated control mice for the pressure overload and MI models, mice underwent the same surgical procedures but without aortic constriction or coronary artery ligation, respectively.

Cardiac function was assessed by transthoracic echocardiography in conscious mice (Aplio SSA-770A, Toshiba Medical Systems Corporation) using a 15-MHz linear-array transducer 1, 4, and 8 weeks after TAC surgery.

### 2.3 Long-term observation

Seven- or eight-week-old male *Fitm1* (−/−), *Fitm2* (+/−), and littermate mice were reared as described above. Cardiac function was assessed by transthoracic echocardiography in conscious mice using a 15-MHz linear-array transducer at the end of approximately two years of observation.

### 2.4 Sample collection, total RNA isolation, and cDNA synthesis

Mice were sacrificed and the left ventricle (LV) was excised. Total RNA was extracted using TRIzol reagent (Life Technologies) and subsequently purified with an RNeasy Mini Kit (Qiagen) following the manufacturer's instructions. mRNA was quantitated using an ND1000 nanodrop spectrophotometer. cDNA was synthesized from 100 ng of total RNA using a QuantiTect Reverse Transcription Kit (Qiagen), in accordance with the manufacturer's protocol.

### 2.5 Gene expression analysis

Quantitative polymerase chain reaction (qPCR) using the TaqMan system (Applied Biosystems) was used to analyze gene expression in TAC and MI samples. TATA-binding protein (Tbp) was used as an internal control. The primers are listed below. Relative mRNA levels were quantified using the comparative ΔΔCT method with Tbp as a reference gene.

*Fitm1* Mm01322192_g1
*Fitm2* Mm04212060_m1
*Tbp* Mm00446971_m1

### 2.6 Statistical analysis

Differences were tested by two-tailed t-test. Values of *p* < 0.05 were considered statistically significant. Data analysis was performed using Microsoft Excel 2010 (Microsoft).

## 3 Results and discussion

**Fig. 1:**
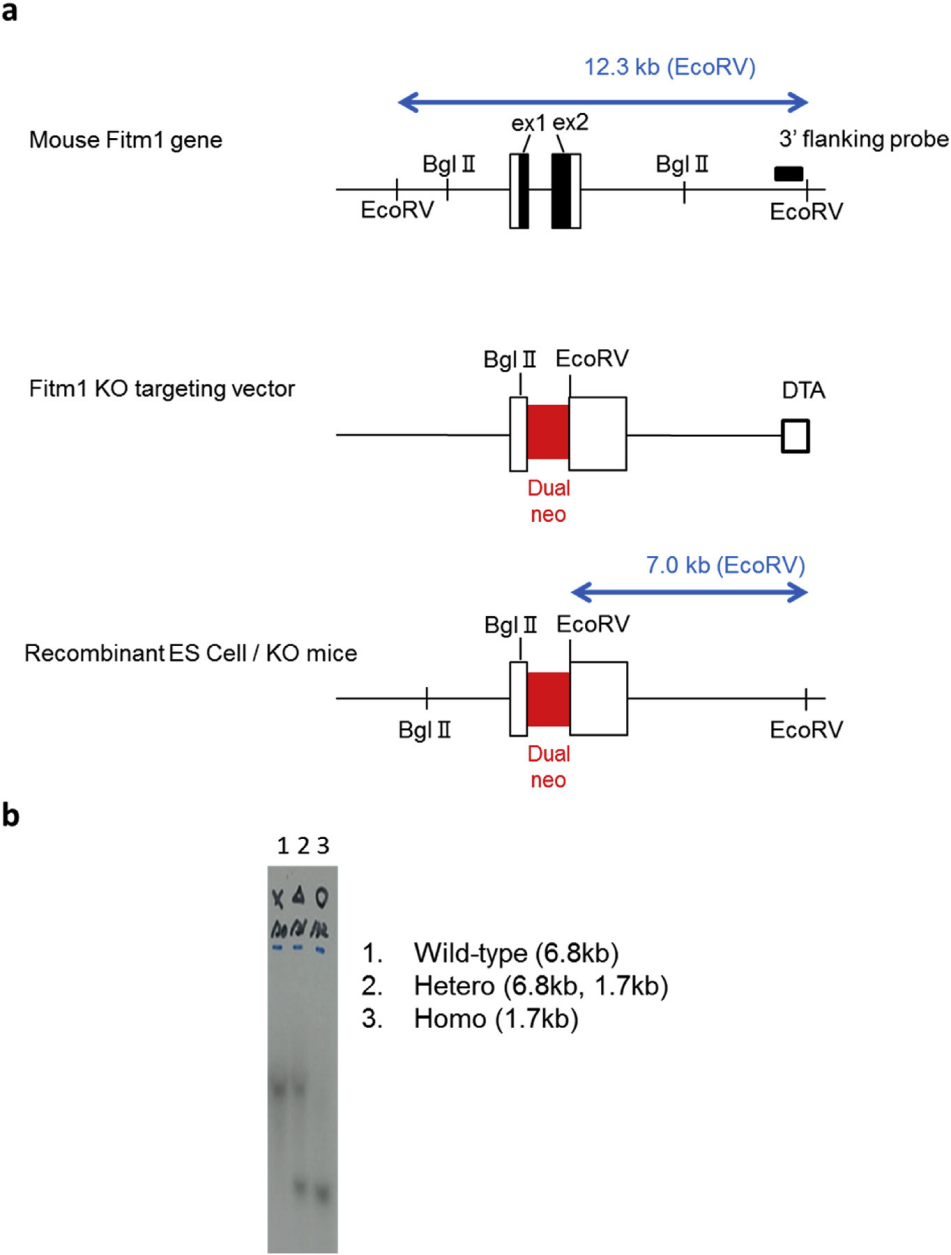
Generation of *Fitm1*-knockout mice by gene targeting. a) Gene-targeting strategy. Top: exon and restriction map of the *Fitm1* gene locus. Middle: targeting vector. In the targeting vector, the neo cassette replaces exon 1 and exon 2 of the *Fitm1* gene. Bottom: targeted allele in embryonic stem cells and knockout (KO) mice. b) Southern blot analysis of genomic DNA from mouse tails. The target allele (∼1.7 kb) was distinguished from the wild-type allele (∼6.8 kb). The wild-type allele was identified in wild-type and heterozygous mice. The target allele was identified in knockout and heterozygous mice.

**Fig. 2:**
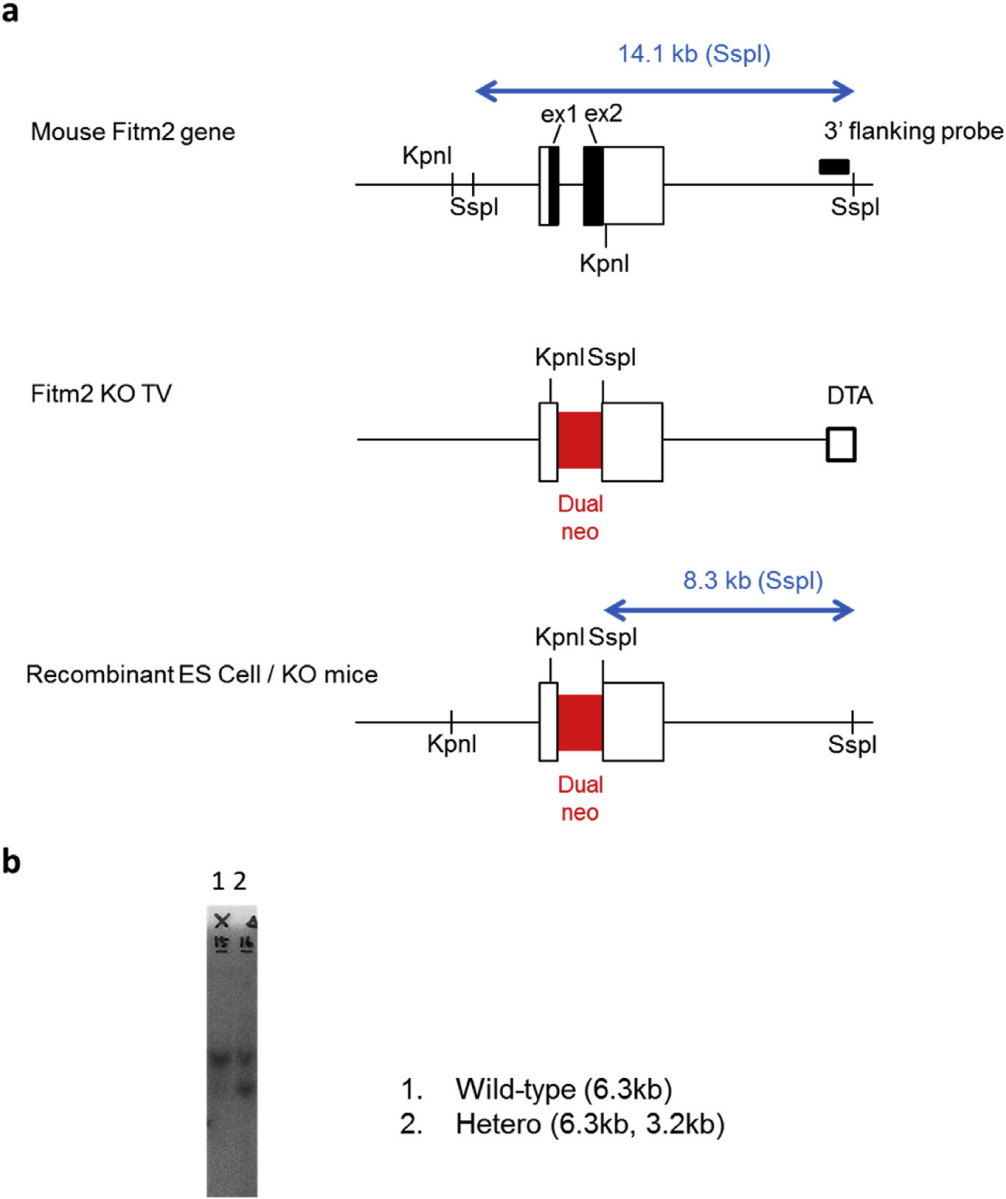
Generation of *Fitm2*-knockout mice by gene targeting. a) Gene-targeting strategy. Top: exon and restriction map of the *Fitm2* gene locus. Middle: targeting vector. In the targeting vector, the neo cassette replaces exon 1 and exon 2 of the *Fitm2* gene. Bottom: target allele in embryonic stem cells and knockout mice. b) Southern blot analysis of genomic DNA from mouse tails. The target allele (∼3.2 kb) was distinguished from the wild-type allele (∼6.3 kb). The wild-type allele was identified in wild-type and heterozygous mice. The target allele was identified in heterozygous mice.

**Fig. 3:**
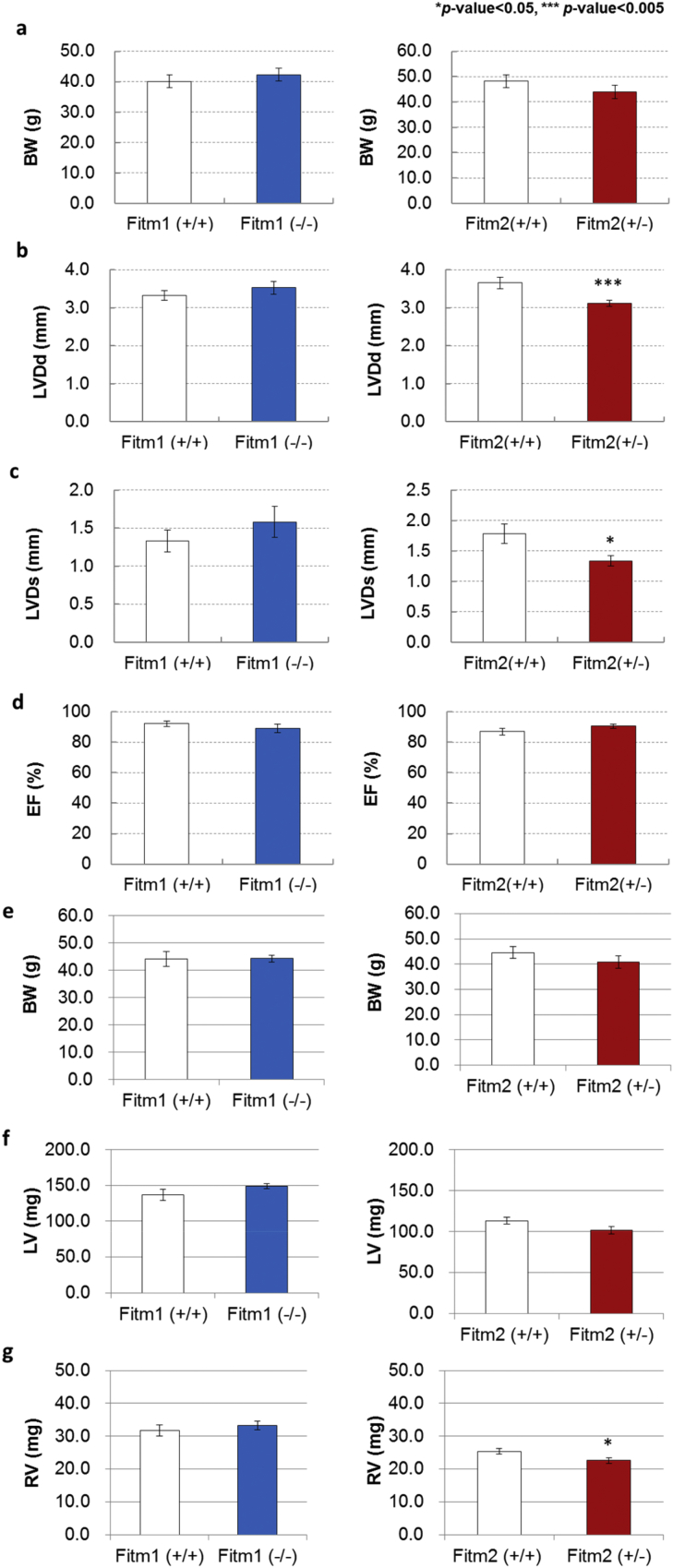
Analysis of the functions of aged hearts. a) Measurements of body weight (BW) at 22 months of age. Error bars indicate SE. b–d) Cardiac function at 22 months of age. Cardiac function was assessed by echocardiography, and mean values for each parameter are shown [*Fitm1* (+/+): *n* = 18, *Fitm1* (−/−): *n* = 17, *Fitm2* (+/+): *n* = 14, *Fitm2* (+/−): *n* = 16]. Error bars indicate SE. e–g) Measurements of BW, LV, and RV at 24 months of age [*n* = 12 in each group of *Fitm1* (+/+) and *Fitm1* (−/−), *n* = 8 in each group of *Fitm2* (+/+) and *Fitm2* (+/−)]. Error bars indicate SE.

To investigate the functions of *Fitm1* and *Fitm2* in the heart, we generated knockout mice using a gene-targeting system (Figs. 1a, 2a). *Fitm1* (−/−) mice were viable, but *Fitm2* (−/−) mice exhibited embryonic lethality (Figs. 1b, 2b), as reported previously [10]. As *Fitm2* (−/−) mice died from embryonic days 9–10 (Nishihama and Nagayama, unpublished data), we used *Fitm1* (−/−) and *Fitm2* (+/−) mice in our experiments. Assessment of cardiac function by echocardiography at 22 months of age showed no differences in body weight (BW), left ventricular end-diastolic dimension (LVDd), left ventricular end-systolic dimension (LVDs), or ejection fraction (EF) between *Fitm1* (−/−) mice and their wild-type littermates [*Fitm1* (+/+)] (Fig. 3a–d). There was no difference in BW, left ventricle (LV) weight, or right ventricle (RV) weight at sacrifice at 24 months of age (Fig. 3e–g). Meanwhile, there was no remarkable difference in BW or EF between *Fitm2* (+/+) and *Fitm2* (+/−) mice. However, LVDd and LVDs were significantly shorter in *Fitm2* (+/−) mice at 22 months of age (Fig. 3a–d). Although *Fitm2* (+/−) mice had lower RV weight than *Fitm2* (+/+) mice, they had similar BW and LV weight (Fig. 3e–g). These results suggest that *Fitm1* deletion does not significantly affect cardiac development or function under normal conditions. Meanwhile, *Fitm2* (+/−) mice exhibited short LVDd and LVDs. However, their EF was normal (Fig. 3b–d) and their survival rate did not differ from that of the wild-type mice (T. Nagayama and N. Nishihama, unpublished data). Thus, under normal conditions, *Fitm2* knockdown does not have a significant impact. However, *Fitm2* (−/−) mice exhibit embryonic lethality, and postnatal whole-body knockout of *Fitm2* leads to lethal enteropathy [7]. These results indicate that *Fitm2* has important roles during development as well as in small intestinal health and function after birth.

**Fig. 4:**
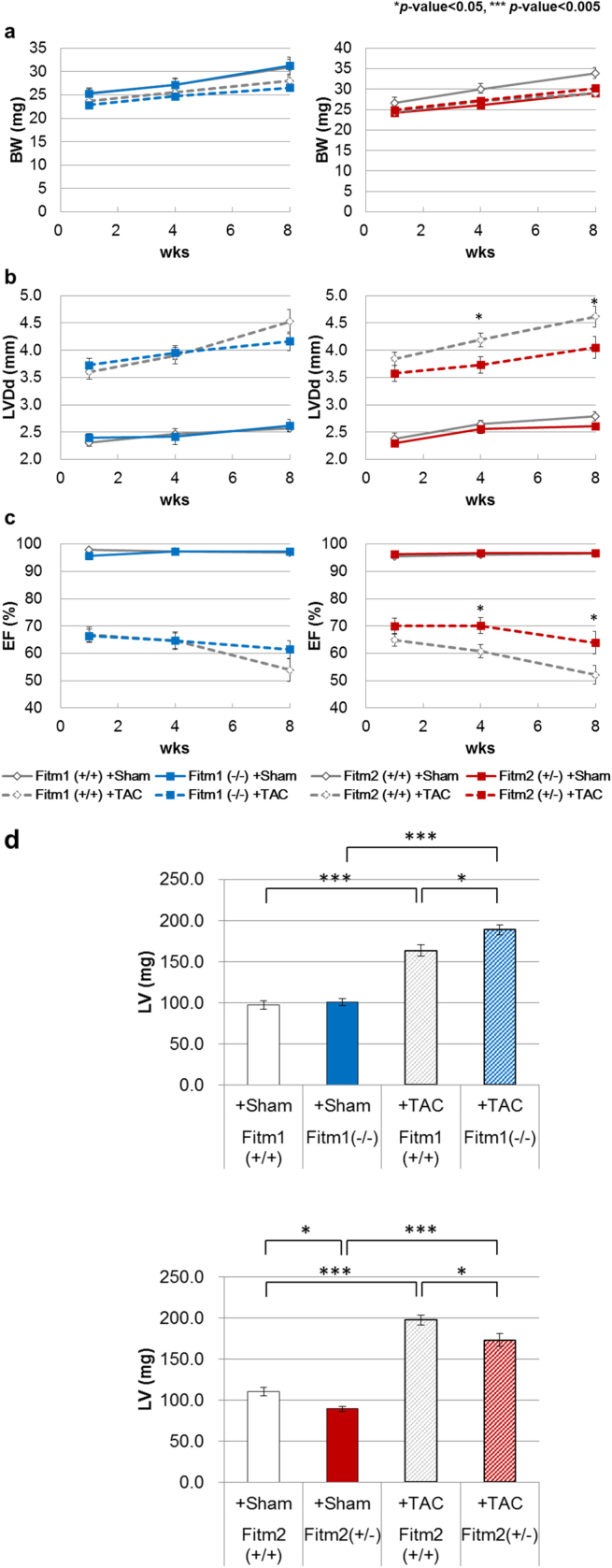
Changes in BW, cardiac function, and LV weight in TAC. *Fitm1* (+/+), *Fitm1* (−/−), *Fitm2* (+/+), and *Fitm2* (+/−) mice were subjected to TAC as described in the Experimental procedures. a–c) Mean values of the indicated parameter in *Fitm1* (+/+), *Fitm1* (−/−), *Fitm2* (+/+), and *Fitm2* (+/−) mice during 8 weeks after TAC [*Fitm1* (+/+)+sham: *n* = 9, *Fitm1* (−/−)+sham: *n* = 8, *Fitm1* (+/+)+TAC: *n* = 34, *Fitm1* (−/−)+TAC: *n* = 44, *Fitm2* (+/+)+sham: *n* = 9, *Fitm2* (+/−)+sham: *n* = 9, *Fitm2* (+/+)+TAC: *n* = 34, *Fitm2* (+/−)+TAC: *n* = 33]. Values are the mean of three experiments. Error bars indicate SE. An asterisk indicates the *p*-value from the comparison between *Fitm2* (+/+)+TAC and *Fitm2* (+/−)+TAC. d) Mean LV weights [*Fitm1* (+/+)-sham: *n* = 9, *Fitm1* (−/−)+sham: *n* = 8, *Fitm1* (+/+)+TAC: *n* = 26, *Fitm1* (−/−)+TAC: *n* = 40, *Fitm2* (+/+)+sham: *n* = 9, *Fitm2* (+/−)+sham: *n* = 9, *Fitm2* (+/+)+TAC: *n* = 25, *Fitm2* (+/−)+TAC: *n* = 27]. Error bars indicate SE.

Next, we investigated whether *Fitm1* or *Fitm2* deletion affects the pathogenesis of heart failure. We induced TAC in *Fitm1* (−/−) and *Fitm2* (+/−) mice and monitored their BW and cardiac function by echocardiography 1, 4, and 8 weeks after TAC surgery. We also monitored the survival rate of the mice subjected to TAC. *Fitm1* (−/−) and *Fitm2* (+/−) mice exhibited increased survival (Tables 1 and 2). The BW of wild-type and *Fitm1* (−/−) mice that underwent TAC decreased from 1 to 8 weeks after surgery. However, the BW of *Fitm2* (+/−) mice that underwent TAC did not differ from that of the sham-operated mice (Fig. 4a; Tables 1 and 2). Within 8 weeks after TAC surgery, wild-type, *Fitm1* (−/−), and *Fitm2* (+/−) mice that underwent TAC developed dilation of the cardiac chamber and contractile dysfunction as measured by LVDd and EF (Fig. 4b, c). There were no significant differences in LVDd or EF between wild-type and *Fitm1* (−/−) mice (Fig. 4b, c). Compared with wild-type mice that underwent TAC, *Fitm2* (+/−) mice exhibited attenuated expanding LV and reduction of EF from 4 weeks after surgery until 8 weeks (Fig. 4b, c).

**Table 1:** Survival rate and tissue weights in response to TAC in *Fitm1* (+/+) and *Fitm1* (−/−) mice.

	Fitm1 (+/+) +TAC	Fitm1 (−/−) +TAC	Fitm1 (+/+) +Sham	Fitm1 (−/−) +Sham	*p*^a^	*p*^b^	*p*^c^	*p*^d^
Survival rate (%)	58.1	72.7	100.0	88.9				
BW (g)	29.2 ± 1.1	27.9 ± 0.8	33.5 ± 2.7	32.7 ± 1.8	NS	NS	<0.05	NS
RV (mg)	36.1 ± 2.8	40.8 ± 2.1	24.7 ± 1.5	25.8 ± 1.1	NS	<0.05	<0.01	NS
LV (mg)	163.5 ± 6.7	188.5 ± 6.4	97.4 ± 5.1	100.3 ± 4.4	NS	<0.005	<0.005	<0.05
Lung (mg)	283.1 ± 25.6	334.6 ± 21.7	155.9 ± 6.4	142.3 ± 5.6	NS	<0.01	<0.005	NS

BW: body weight, RV: right ventricle, LV: left ventricle, NS: not significant.

*Fitm1* (+/+) and *Fitm1* (−/−) mice were subjected to TAC as described in the experimental procedures. The survival rate and mean weights of tissues from *Fitm1* (+/+) and *Fitm1* (−/−) sham- or TAC-operated mice are shown [*Fitm1* (+/+)+sham: *n* = 9, *Fitm1* (−/−)+sham: *n* = 8, *Fitm1* (+/+)+TAC: *n* = 26, *Fitm1* (−/−)+TAC: *n* = 40].

a *Fitm1* (−/−)+sham vs. *Fitm1* (+/+)+sham.

b *Fitm1* (+/+)+TAC vs. *Fitm1* (+/+)+sham.

c *Fitm1* (−/−)+TAC vs. *Fitm1* (−/−)+sham.

d *Fitm1* (+/+)+TAC vs. *Fitm1* (−/−)+TAC.

**Table 2:** Survival rate and tissue weights in response to TAC in *Fitm2* (+/+) and *Fitm2* (+/−) mice.

	Fitm2 (+/+) +TAC	Fitm2 (+/−) +TAC	Fitm2 (+/+) +Sham	Fitm2 (+/−) +Sham	p^a^	p^b^	p^c^	p^d^
Survival rate (%)	54.5	65.9	100.0	100.0				
BW (g)	31.1 ± 1.4	32.8 ± 1.0	37.3 ± 2.3	31.7 ± 1.7	NS	<0.05	NS	NS
RV (mg)	37.2 ± 3.0	34.8 ± 2.7	24.4 ± 1.4	20.4 ± 1.1	<0.05	<0.05	<0.01	NS
LV (mg)	197.3 ± 6.2	173.1 ± 7.9	109.9 ± 5.2	89.2 ± 2.9	<0.005	<0.005	<0.005	<0.05
Lung (mg)	302.0 ± 29.6	280.9 ± 28.8	172.7 ± 12.7	140.8 ± 4.5	<0.05	<0.05	<0.01	NS

BW: body weight, RV: right ventricle, LV: left ventricle, NS: not significant.

*Fitm2* (+/+) and *Fitm2* (+/−) mice were subjected to TAC as described in the experimental procedures. The survival rate and mean weights of tissues from *Fitm2* (+/+) and *Fitm2* (+/−) sham- or TAC-operated mice are shown [*Fitm2* (+/+)+sham: *n* = 9, *Fitm2 (+/−*)+sham: *n* = 9, *Fitm2* (+/+)+TAC: *n* = 25, *Fitm2* (+/−)+TAC: *n* = 27].

a *Fitm2* (+/−)+sham vs. *Fitm2* (+/+)+sham.

b *Fitm2* (+/+)+TAC vs. *Fitm2* (+/+)+sham.

c *Fitm2* (+/−)+TAC vs. *Fitm2* (+/−)+sham.

d *Fitm2* (+/+)+TAC vs. *Fitm2* (+/−)+TAC.

After measuring cardiac function, we dissected the mice and weighed their LV, RV, and lungs. The LVs from *Fitm1* (+/+) TAC mice were approximately twice as heavy as those from the sham-operated mice (Fig. 4d, Table 1); these results are consistent with the cardiac dysfunction described above (Fig. 4b, c). Furthermore, the RV of *Fitm1* (+/+) and *Fitm1* (−/−) TAC mice was approximately 1.5 times as heavy as that of the sham-operated mice (Table 1). There were no significant differences in RV weight between *Fitm1* (+/+) TAC mice and *Fitm1* (−/−) TAC mice, and the LV of *Fitm1* (−/−) TAC mice was heavier than that of *Fitm1* (+/+) TAC mice (Fig. 4d, Table 1). The lungs from *Fitm1* (+/+) TAC mice and *Fitm1* (−/−) TAC mice were approximately 2.5 times heavier than those from the sham-operated controls (Table 1); these results are consistent with the results regarding pulmonary congestion described above. However, there was no significant difference in lung weight between *Fitm1* (+/+) TAC mice and *Fitm1* (−/−) TAC mice.

The tissue weights of *Fitm2* (+/+) and *Fitm2* (+/−) mice were greater in mice that underwent TAC than in those that did not (Fig. 4d, Table 2), reflecting their condition. The LV weight of *Fitm2* (+/−) TAC mice was increased compared with that of the sham-operated mice; however, it is a smaller extent than that of *Fitm2* (+/+) TAC mice (Fig. 4d). These results might be consistent with the suppression of dilation (Fig. 4b, c). Although the LV from *Fitm2* (+/−) mice that did not undergo TAC weighed less than that of wild-type mice, there were no differences between groups with respect to LVDd or EF (Fig. 4b, c). There was also no significant difference in lung weight between *Fitm2* (+/+) TAC mice and *Fitm2* (+/−) TAC mice (Table 2).

**Fig. 5:**
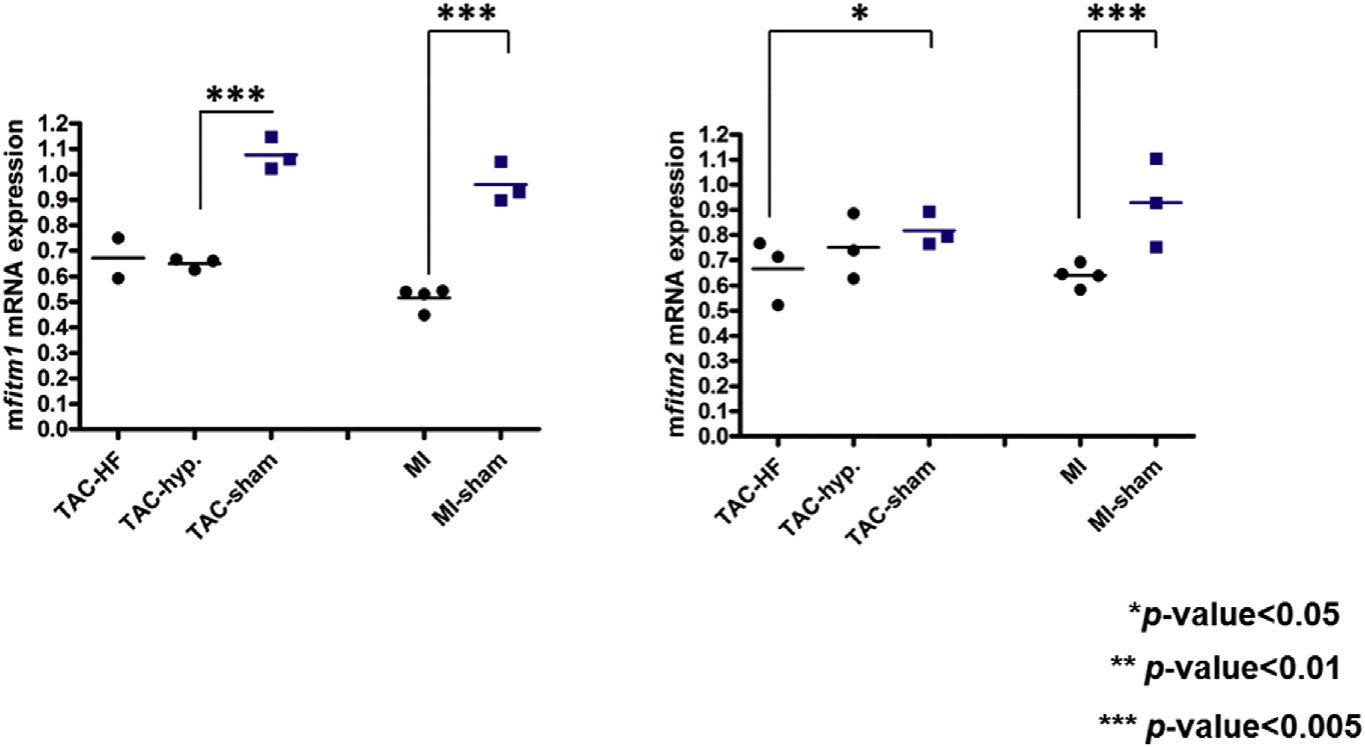
Quantitative analysis of mRNA expression in hearts from TAC and MI model mice. Relative mRNA levels were quantified using the comparative ΔΔCT method with an internal control gene (*Tbp*). Each dot represents an individual animal. *Fitm1* mRNA expression was reduced ∼0.48-fold (*p*-value < 0.005) in TAC-induced hypertrophy, ∼0.62-fold (*p*-value < 0.005) in TAC-induced heart failure, and ∼0.54-fold (*p*-value < 0.005) in MI-induced heart failure. *Fitm2* mRNA expression was also significantly reduced in both TAC-induced heart failure (∼0.76-fold, *p*-value < 0.05) and MI-induced heart failure (∼0.69-fold, *p*-value < 0.005), and showed a trend of reduction in TAC-induced hypertrophy, although the difference was not significant.

Together with the cardiac function data, our findings suggest that deletion of *Fitm1* improves mouse survival in the TAC model without altering the pathological condition. On the other hand, *Fitm2* (+/−) positively affects cardiac function and survival after pressure overload-induced heart failure. The different responses of *Fitm1* (−/−) and *Fitm2* (+/−) to TAC are thought to reflect their tissue expression patterns. *Fitm1* mRNA expression is higher in skeletal muscle than in the heart, whereas *Fitm2* mRNA expression shows the opposite pattern [1]. Our data suggest that deletion of FITM2 has protective effects against heart failure. Moreover, we found that the mRNA expression of both *Fitm1* and *Fitm2* was decreased in model mice with chronic heart failure induced by pressure overload (TAC model) and myocardial infarction (MI model) (Fig. 5), suggesting that *Fitm1* and *Fitm2* are common response genes in heart failure due to volume and pressure overload. It would be interesting to analyze the effects of their deletion in chronic heart failure induced by MI, and the mechanistic insights together with that in TAC model. Previously, we found that Fitm1 were identified by proteomic analysis of endoplasmic reticulum/sarcoplasmic rich fraction of mice heart, but there was no significant difference between TAC-operated and sham mice, probably because, the protein expression changed little as a result of TAC [9]. Therefore, it also would be important to analyze the detailed protein expression pattern in both heart failure model.

In this study, we mainly analyzed the effects of *Fitm1* and *Fitm2* deletion in healthy and diseased heart by gravimetric analysis and echocardiography. Therefore, further studies are needed to investigate the molecular mechanisms behind the observed protective effects against heart failure especially by *Fitm2* deletion, such as, whether structural changes are occurred in heart and cardiac cells of knockout mice. Determination of functional changes of cardiac cells of knockout mice is also informative. Fitm1 and Fitm2 are involved in lipid droplet formation [1, 2, 3], though relationship between lipid droplet accumulation and pathological condition of heart failure is still unknown, it is known that lipid content in myocardiocytes is increased in failing hearts, especially in patients with diabetes mellitus and the metabolic syndrome [11]. It also would be interesting to determine lipid droplet number and size in tissues of knockout mice and analyze further mechanistic insights. In the view of the energy metabolism, heart failure is accompanied by reduced fatty acid utilization and ATP level [12, 13, 14]. Mice with skeletal muscle-specific overexpression of *Fitm2* are reported to exhibit decreased fatty acid oxidation, increased utilization of branched-chain amino acids, enhanced glucose uptake, and decreased cellular ATP levels in skeletal muscle [8]. These findings suggest that the overexpression of *Fitm2* in mice might alter cellular metabolism in a manner similar to the changes observed in heart failure conditions. Nevertheless, in this study, we have not evaluated the products of energy metabolism of *Fitm1* (−/−) or *Fitm2* (+/-) mice in the context of healthy condition and heart failure. Whether metabolic changes are occurred in knockout mice is an important question to be addressed. Considering that *Fitm1* and *Fitm2* are family genes, it would be important to investigate whether compensate pathway to each other is occurred in knockout mice. Furthermore, analysis of *Ftim1* and *Fitm2* double knockout mice will also be informative to determine their functions in heart and heart failure.

Pharmacological agents generally cannot completely inhibit protein expression or function. Thus, considering our results from knockout mice that underwent TAC, pharmacological downregulation or inhibition of FITM2 may contribute to the treatment of heart failure without having negative effects in otherwise healthy conditions.

## Declarations

### Author contribution statement

Natsumi Nishihama: Conceived and designed the experiments; Performed the experiments; Analyzed and interpreted the data; Contributed reagents, materials, analysis tools or data; Wrote the paper.

Takahiro Nagayama: Conceived and designed the experiments; Performed the experiments; Analyzed and interpreted the data; Contributed reagents, materials, analysis tools or data.

Shinji Makino: Analyzed and interpreted the data.

Ryuta Koishi: Conceived and designed the experiments; Analyzed and interpreted the data.

### Funding statement

This work was supported by Daiichi Sankyo Co., Ltd.

### Competing interest statement

The authors declare the following conflict of interest: Natsumi Nishihama, Takahiro Nagayama, and Ryuta Koishi are employees of Daiichi Sankyo Co., Ltd.

### Additional information

No additional information is available for this paper.
